# The *Wukong* Terminal-Repeat Retrotransposon in Miniature (TRIM) Elements in Diverse Maize Germplasm

**DOI:** 10.1534/g3.115.018317

**Published:** 2015-05-26

**Authors:** Zhen Liu, Xinxin Li, Tingzhang Wang, Joachim Messing, Jian-Hong Xu

**Affiliations:** * Institute of Crop Science, Zhejiang Key Laboratory of Crop Germplasm, Zhejiang University, Hangzhou, Zhejiang 310058, China; † Waksman Institute of Microbiology, Rutgers, The State University of New Jersey, Piscataway, New Jersey 08854

**Keywords:** TRIM, retrotransposon, insertion polymorphism, NGS, maize (*Zea mays*)

## Abstract

TRIMs (terminal-repeat retrotransposons in miniature), which are characterized by their small size, have been discovered in all investigated vascular plants and even in animals. Here, we identified a highly conservative TRIM family referred to as *Wukon*g elements in the maize genome. The *Wukong* family shows a distinct pattern of tandem arrangement in the maize genome suggesting a high rate of unequal crossing over. Estimation of insertion times implies a burst of retrotransposition activity of the *Wukong* family after the allotetraploidization of maize. Using next-generation sequencing data, we detected 87 new *Wukong* insertions in parents of the maize NAM population relative to the B73 reference genome and found abundant insertion polymorphism of *Wukong* elements in 75 re-sequenced maize lines, including teosinte, landraces, and improved lines. These results suggest that *Wukon*g elements possessed a persistent retrotransposition activity throughout maize evolution. Moreover, the phylogenetic relationships among 76 maize inbreds and their relatives based on insertion polymorphisms of *Wukong* elements should provide us with reliable molecular markers for biodiversity and genetics studies.

Retrotransposons are widespread and can constitute a major portion of eukaryotic genomes; they could account for approximately 85% in the genome of some plant species (Schnable *et al.* 2009; Huang *et al.* 2012). Retrotransposons mainly fall into two classes: long terminal repeats (LTR) and non-LTR retrotransposons based on the present or absent of LTRs (Wicker *et al.* 2007). LTR retrotransposons, including Ty1-copia and Ty3-gypsy, are predominant in plants, whereas non-LTR retrotransposons, such as LINEs and SINEs, are more abundant in animals (Grandbastien 1992; Kumar and Bennetzen 1999). They also can be divided into autonomous and nonautonomous retrotransposons. The autonomous retrotransposons contain polyproteins (at least the *gag* and *pol* coding regions) for retrotransposition, whereas nonautonomous retrotransposons lack functional polyproteins and their mobilization depends on the *trans*-acting function of the corresponding autonomous elements (Kajikawa and Okada 2002; Dewannieux *et al.* 2003). Furthermore, there are two groups of nonautonomous LTR retrotransposons, large retrotransposon derivatives (LARD) and terminal repeat retrotransposons in miniature (TRIM) (Witte *et al.* 2001; Kalendar *et al.* 2004). They differ in the loss of their moving ability that resulted from mutations of coding domains residing in LARDs and the deletion of internal coding region in TRIMs. Because they amplify by a copy-and-paste mechanism, they remain inserted at their insertion sites and, therefore, each retrotransposon family is dispersed in a range of copy numbers throughout the genome.

TRIM elements typically possess short overall length (<540 bp), which consists of two 100- to 250-bp terminal direct repeats (TDRs) or LTRs and an internal domain of 100 to 300 bp (Witte *et al.* 2001). To date, they have been discovered in all investigated vascular plants and even in animals. Because they are similar to SINEs and MITEs in their short size, TRIMs could be useful molecular markers for investigating evolutionary relationships and genetic diversity in plants (Antonius-Klemola *et al.* 2006; Yang *et al.* 2007). It has been reported that TRIMs also seem to be involved actively in altering gene structure, regulating gene expressing, reshaping genomes, and mediating horizontal transfer of DNA (Witte *et al.* 2001; Yang *et al.* 2007; Zhou and Cahan 2012).

As an economically important crop and model organism for biological research, maize (*Zea mays* ssp. *mays*) is thought to have originated from its wild relative, Balas teosinte (*Z. mays* ssp. *parviglumis*), approximately 9000 years ago in southern Mexico (Matsuoka *et al.* 2002). Maize underwent two distinct selections: domestication and crop improvement. Landraces have arisen due to the accumulation of mutations as well as differential fixation of standing variation in the teosintes prior to domestication, whereas inbred lines were selected from landraces to enable breeding for hybrid vigor (Yamasaki *et al.* 2005). Since the first transposon had been identified in maize (McClintock 1950), almost all classes of TE have been discovered in maize, which constitute up to 85% of the maize genome, and they generally are dispersed throughout the whole genome (Schnable *et al.* 2009). Still, there is little information on TRIM elements in maize so far. However, with next-generation sequencing (NGS) technology it becomes possible to identify genetic variations on a genome-wide scale, such as single nucleotide polymorphisms (SNP), small insertions/deletions (Indels), and several classes of structural differences (Korbel *et al.* 2007; Nielsen *et al.* 2011). Furthermore, the NGS short reads have been successfully applied to obtain the complete profiles of TE insertion variations in human, rice, maize, and soybean (Naito *et al.* 2009; Hormozdiari *et al.* 2010; Ewing and Kazazian 2011; Tian *et al.* 2012; David *et al.* 2013) .

Here, we identified a TRIM retrotransposon in maize named *Wukon*g and found that they are frequently present in the form of tandem arrays. Furthermore, a large number of *Wukong* insertions were identified in the parents of maize NAM populations (McMullen *et al.* 2009) using NGS data, and the phylogenetic relationship of three germplasm groups, including teosinte, landraces, and inbred lines, further revealed insertion polymorphisms of *Wukong* elements. Additionally, a putative autonomous counterpart of *Wukong* elements can be proposed because of a conserved 12-bp binding site in their LTRs.

## Materials and Methods

### Plant materials

B73 inbred and 25 parents of the maize NAM population were kindly provided by North Central Regional Plant Introduction Station (NCRPIS): B97, CML103, CML228, CML247, CML277, CML322, CML333, CML52, CML69, Hp301, IL14H, Ki11, Ki3, Ky21, M162W, M37W, Mo18W, MS71, NC350, NC358, Oh43, Oh7B, P39, Tx303, and Tzi8.

### Genome sequences

The maize B73 reference genome sequences (RefGen_V2) were downloaded from maizesequence (http://archive.maizesequence.org/index.h​tml). The NGS data of 75 genomes of maize and its wild relatives from the Maize Hapmap2 project (Chia *et al.* 2012) were downloaded from the NCBI Short Read Archive (Accession: SRA051245). The sequences of chromosomal regions of the *z1C1* locus from B73 (AC144717, AC144718) and BSSS53 (AF528565, AF090447) were downloaded from NCBI GenBank.

### Genome-wide identification of *Wukong* elements

The tBlastN program was used to search against the maize B73 reference genome with the sequence of *Wukong* as a query to identify *Wukong* elements, and sequence alignments were manually validated.

The nucleotide sequences of TSDs and their 5-bp flanking sequences at both sides served as the query to identify their positions in the genome. A total of 118 elements with intact TSD, including 90 complete TRIM elements and 28 solo-LTRs, were used to generate the sequence logo with Weblogo 3 (http://weblogo.threeplusone.com).

### Insertion time estimation

Following an empirically derived formula (Sanmiguel *et al.* 1998), we used nucleotide divergence of the left and the right LTR of each intact element to determine the relative insertion times of *Wukong* elements. Specifically, both LTRs of the complete elements were aligned by CLUSTALW2 with default parameters. Then, these aligned sequences were used to calculate the average number of nucleotide substitutions per site (K) using MEGA v5.0 with the Kimura-2 parameter model. Finally, the insertion times (T) of the intact *Wukong* elements were measured with the equation T = K/2r, where r measures an average nucleotide substitution rate, which has been set to 1.3×10^−8^ substitutions per site per year, as proposed previously (Ma and Bennetzen 2004).

### PCR and reverse-transcript (RT) PCR amplifications

Genomic DNAs from B73 and 25 parents of NAM population were extracted from 2-wk seedlings using standard cetyltrimethyl ammonium bromide (CTAB) methods, and three *Wukong* elements were randomly selected to validate the insertion sites by PCR amplification. Three pairs of primers were designed by Primer v5.0 (Supporting Information, Table S1). PCR reaction was conducted in 20 μl volume solution, including 50 ng of genomic DNA, 10 pmol of each primer, 1× PCR buffer, 250 μM dNTPs, and 1.0 unit Ex Taq DNA polymerase. The cycling conditions were 95° for 2 min, 35 cycles of 95° for 15 sec, 56° for 30 sec, and 72° for 40 sec, followed by a final extension at 72° for 5 min. PCR products were analyzed by agarose gel electrophoresis stained with ethidium bromide (EB).

Total RNA of 2-wk seedlings and pollen of B73 were isolated with TRIzol reagent (Invitrogen, USA) according to the instructions of the manufacturer and then treated with RNase-free DNaseI (Fermentas, USA) to remove contaminating genomic DNA. Go Script Reverse Transcription System (Promega, USA) was used to prepare cDNA from treated total RNA. Finally, PCR reaction with cDNA as templates was performed as stated previously. The β-actin gene was used as a control to correct the concentration of cDNAs among different samples (Table S1).

### Identification of *Wukong* elements and insertion variation by NGS data

The NGS data of 75 genomes of the maize Hapmap2 project contain pair-end reads ranging from 132 to 250 bp in length. *Wukong* elements were identified with the following steps. First, short reads were aligned to the LTR sequence and reads containing one of the two ends of LTRs and flanking genomic sequences were selected. Reads aligned only to LTRs in the absence of flanking sequences were excluded due to the lack of positional information. Second, the selected reads were mapped to the B73 reference genome and only reads mapped to a unique location in the genome were retained. Third, the sequences near the mapped locations were manually inspected.

After identifying *Wukong* elements from NGS data, the 150-bp sequences immediately flanking the insertion sites were extracted from the B73 reference genome and grouped into a database, which were used as queries to search NGS short reads of 76 resequenced accessions one by one; alignments were trimmed to retain the matching portions and then were extracted from the corresponding databases. The remaining sequences of the reads were manually compared with the sequences of TRIM LTRs to discern whether these locations possess the expected TRIM insertions.

### Phylogenetic analysis

To facilitate the study of genetic relationships and population structure among accessions, we transferred the insertion polymorphism of TRIMs to the form of a data matrix, where “1” and “0” mean the presence or absence of TRIMs, respectively, whereas a question mark represents missing data at given sites (Cheng *et al.* 2003). PAUP* 4.0b10 (Swofford 2002) was used to construct the phylogenetic tree for all accessions based on the data matrix. The Mean model (the mean of pairwise character difference), which can adjust for missing data in the matrix, was used to calculate the pairwise distance. Finally, the Neighbor-Joining method was applied to construct the tree with the least-squares option.

STRUCTURE version 2.1 (Pritchard *et al.* 2000) was used to assess the genetic structure of populations. During this process, the K value, which represents the assumed number of populations, was designated to range from one to seven, and for each K value more than five replicates were performed. A burn-in period of 100,000 iterations followed by a run length of 100,000 iterations, the admixture model, and the correlated allele frequencies model were set. DISTRUCT program was used to graphically display the results (Rosenberg 2004).

## Results

### Identification, characterization, and distribution of *Wukong* elements in the genome

**Figure 1:**
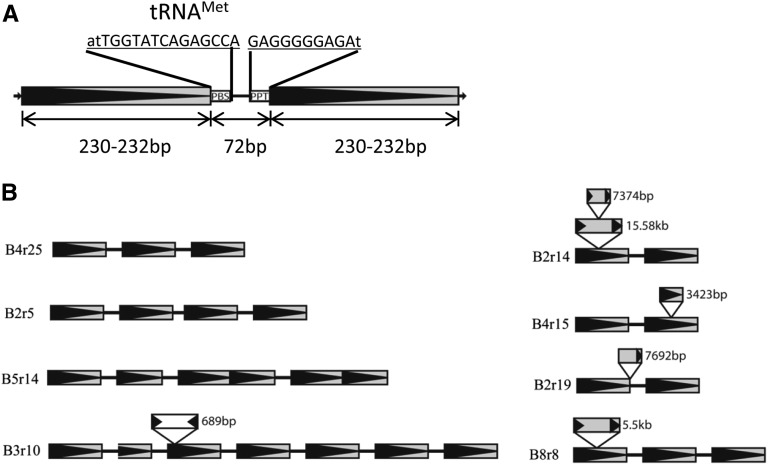
Structures of the *Wukong* elements. (A) Schematic diagram of the general structure of *Wukong* element. (B) The representative *Wukong* elements with tandem duplication and transposable elements insertion.

When aligning the nucleotide sequences of the *z1C1* region, a cluster of the 22-kD zein storage protein genes on maize chromosome 4S between B73 and BSSS53 (Song and Messing 2003), a small LTR retrotransposon was identified. Further analysis revealed that this retrotransposon possesses the typical characteristics of a TRIM element with length of 532 bp, two 230-bp LTRs, and a 72-bp internal domain, which contains two conserved motifs (PBS and PPT) required for transcription (Figure 1A), which was named *Wukong*.

To investigate the abundance of *Wukong* elements in the genome, tBlastN was performed against the sequenced maize B73 genome using the sequences of the *Wukong* element as a query. A total of 215 members were identified, including 89 intact, 28 solo, and 98 truncated elements (Table S2). The majority of the intact and solo elements have 5-bp target site duplications (TSD) (Table S2). In addition, the majority of the elements shared up to 90% identity of their LTRs, internal domains, and even overall sequences, which suggested a series of recent retrotransposition events of *Wukong* elements.

**Figure 2:**
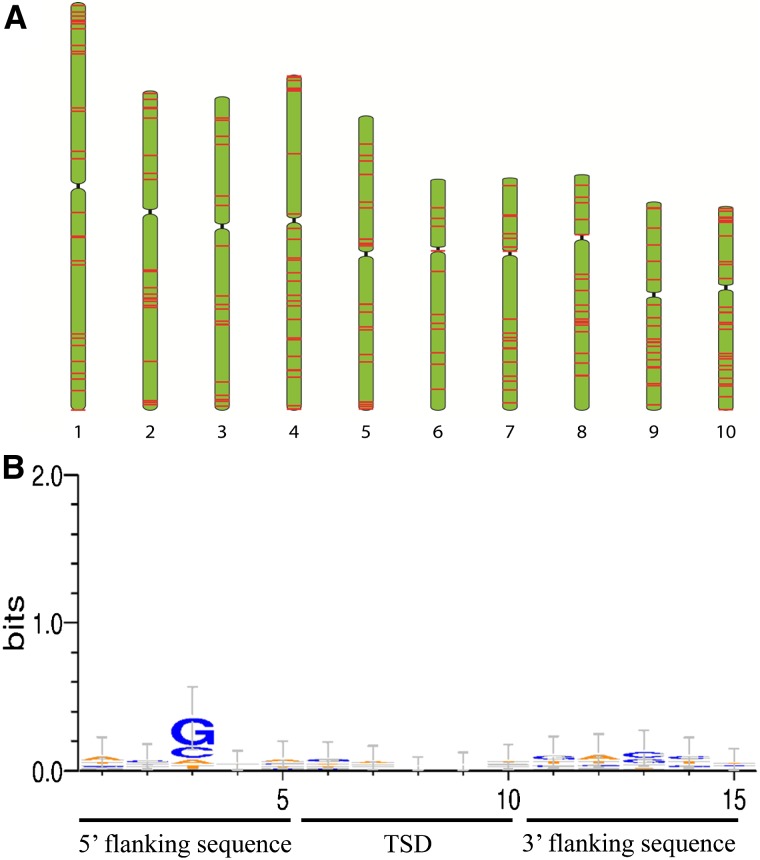
Distribution of *Wukong* elements in the maize genome. (A) Chromosomal distribution of *Wukong* elements in maize B73 genome. (B) Analysis of insertion site preference. TSDs along with the flanking sequences from 90 complete TRIM elements and 28 solo LTRs were used to generate the sequence logo.

The properties of target sites of *Wukong* elements were investigated by analyzing the 5-bp long TSDs and their 5-bp flanking sequences of a total of 122 *Wukong* elements including 71 intact and 28 solo elements. However, there appeared to be no bias of insertion sites, suggesting a rather random distribution of elements in the maize genome (Figure 2B).

A number of mobile elements including DNA transposons and retrotransposons preferentially integrated into pericentromeric regions relative to chromosomal arms (Jiang *et al.* 2002; Rizzon *et al.* 2002; Lippman *et al.* 2004; Paterson *et al.* 2009; Schmutz *et al.* 2010). However, all 215 identified *Wukong* elements were spread through all 10 chromosomes (Figure 2A). Still, 27 members mapped to chromosome 10, yielding the highest density of elements in the genome (0.18 per Mb, 27/143 Mb), whereas only 12 were located on chromosome 6 with the lowest density of elements (0.07 per Mb, 12/161 Mb) (Table S3). Other genomic regions frequently contained a variable number of elements even within the same chromosome (Figure 2A).

### Tandem arrangement and nested insertion

Of the 89 intact *Wukong* elements, eight contain three LTRs, five contain four LTRs, and two elements contain six and seven LTRs, suggesting a distinct pattern of arrangement in the maize genome (Figure 1B). Furthermore, the LTRs usually were arranged in the same orientation within the element with the internal region intact. Such an arrangement suggested that these elements were generated from the recombination events between intact elements, similar as in the case of solo LTR. In addition, a small portion of elements (11%) revealed insertions of other transposable elements (Table S2). For instance, the B3r10 element contained an insertion of 689-bp DNA transposon of the *hAT* family, whereas B8r8 had a 5.5-kb copia-like LTR retrotransposon insertion (Figure 1B).

### Insertion times of *Wukong* elements

**Figure 3:**
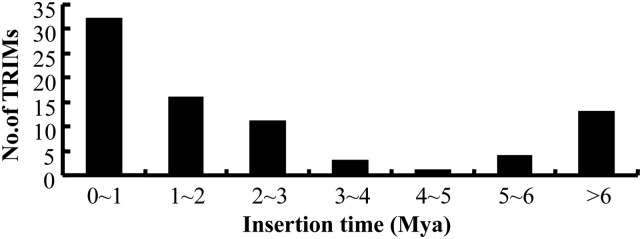
Frequency distribution of the estimated age of 81 intact *Wukong* elements from the maize B73 genome.

Given the sequence divergences between two LTRs of intact elements, we determined the insertion times of 80 intact *Wukong* elements in the B73 genome, which were estimated to range from 0 to 11 million years (Figure 3). Sixty-three (80%) were inserted into the genome within the past five million years, and the distribution of insertion times showed an accelerating trend within this time frame. Thirty-two elements (40%) were integrated into the genome even within the past one million years. These results implied relatively high transposition activity of *Wukong* elements since the transition from tetraploidy to diploidy of maize genome, which occurred approximately 4.8 MYA (Swigonova *et al.* 2004). In addition, three *Wukong* elements (B9r4, B9r7, B9r19) have two identical LTRs, suggesting that the *Wukong* element family could still be active recently. Comparison of orthologous regions of the two progenitors of maize and rice genomes showed an uneven expansion in genic regions with Ty1/copia-like elements rather than Ty3/gypsy-like elements after allotetraploidization, which could also explain the distribution of *Wukong* elements in the maize genome (Bruggmann *et al.* 2006).

### *Wukong* elements in the parents of the NAM population

The nested association mapping (NAM) population contains 25 diverse lines (25DL) across today’s maize germplasm (McMullen *et al.* 2009), which was used to facilitate the maize Hapmap2 project by generating NGS data from 103 inbred lines including the NAM population (Chia *et al.* 2012). Taking advantage of the data, a total of 196 *Wukong* elements were identified in 25 parents of the NAM population with unique matches from approximately 350 Gb of resequencing data. All elements were then mapped to the B73 reference genome, and 87 *Wukong* elements, which were not present in B73, were identified (Table S4). Furthermore, out of 87 elements, 22 (25%) were present in multiple lines, 13 (15%) were in two lines, and 52 (60%) were line-specific (Table S4), suggesting that *Wukong* elements maintained their activity at least during the process of crop improvement.

**Figure 4:**
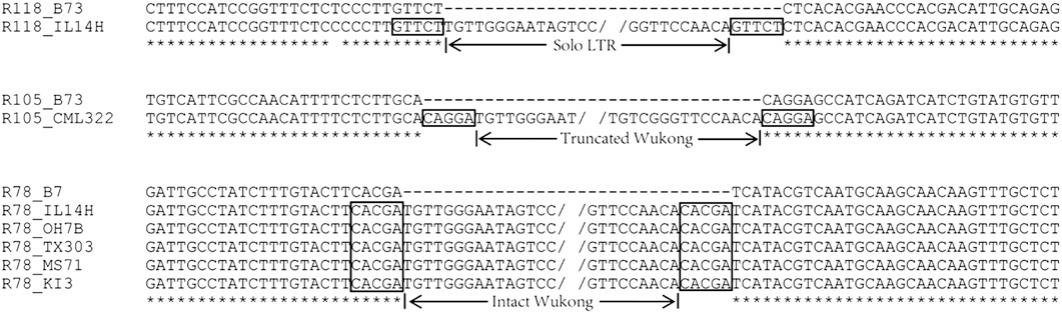
Sequencing validation of three nonreference *Wukong* elements detected with the NGS data from the NAM parents. The R118 site contains a solo LTR in IL14H; the R105 site contains a 170-bp truncated *Wukong* in CML322; and the R78 site contains an intact *Wukong* member in IL14H, OH7B, TX303, MS71, and KI3. TSDs are boxed and identical sites are denoted by an asterisk.

To assess the reliability of the results obtained from NGS data, three representative nonreference *Wukong* elements detected by NGS reads were selected to be subjected to PCR amplification and sequencing. One element (R78) detected from five NAM parents were also validated by PCR and sequencing. The results showed that all these sites contain *Wukong* insertions in the corresponding lines, whereas B73 has none of them (Figure 4).

### Insertion polymorphism of *Wukong* elements in maize and their wild relatives

Given the recent activity of *Wukong* elements in diverse maize germplasm, the question can be raised whether they could serve as molecular markers. Therefore, we investigated the presence or absence of them in 75 resequenced maize and teosinte lines from three germplasm groups composed of 35 modern maize lines, 23 landraces, and 17 teosintes (Hufford *et al.* 2012). Using NGS data, we examined the insertion patterns of a total of 80 *Wukong* members, including 35 insertions absent in B73 and 45 insertions present in all strains (Table S5). A total of approximately 20,000 reads containing the sequences immediately flanking *Wukong* insertion sites in the genome were identified from 781 Gb of resequencing data. For each examined site there are, on average, three short reads supporting the presence or absence of *Wukong* elements. On average, 82% (5038/6156) of putative insertion sites could be predicted with current genome coverage of the population. However, it is noted that the teosinte group has the lowest proportion of detectable sites with an average of 75% in comparison to modern lines and landrace groups with an average of 84% and 85%, respectively (Table S5).

Of 80 *Wukong* elements, 65 (Table S5, column 16–80) were predicted in all three germplasm groups, including 30 (Table S5, column 51–80) insertions present in all 76 accessions, suggesting that their retrotransposition occurred before maize domestication; 11 (Table S5, column 5–15) were detected only in the landrace and modern lines group, indicating that they were presumably activated during domestication; four (Table S5, column 1–4) were unique to modern lines, showing that their insertion presumably happened during crop improvement. In addition, given that the 25 parents of the NAM population were classified into two subgroups, tropical subgroup and temperate subgroup, we found that four *Wukong* elements (Table S5, column 15–18) were specifically present in the tropical subgroup, suggesting that these *Wukong* elements had a unique amplification in tropical improved lines relative to the others. These implied that *Wukong* elements not only possessed a persistent retrotransposition activity throughout maize evolution but also could be used as molecular markers for biodiversity and genetics studies.

**Figure 5:**
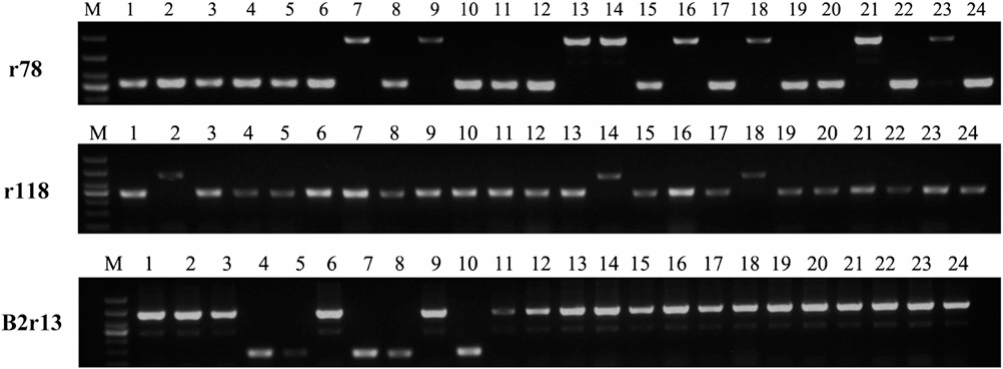
PCR analysis for the detection of insertion polymorphism across the parents of the NAM population. The order of lines denoted by lanes 1–24 is the same as in Table S1. M represents molecular marker.

Similar to a previous analysis, we randomly selected three *Wukong* sites, including two insertions absent in B73 and one B73 insertion, for PCR amplification to confirm the presence or absence in the parents of the NAM population (Figure 5). The PCR results were consistent with NGS data.

### Phylogenetic analysis of TRIM insertion polymorphisms in diverse germplasms

**Figure 6:**
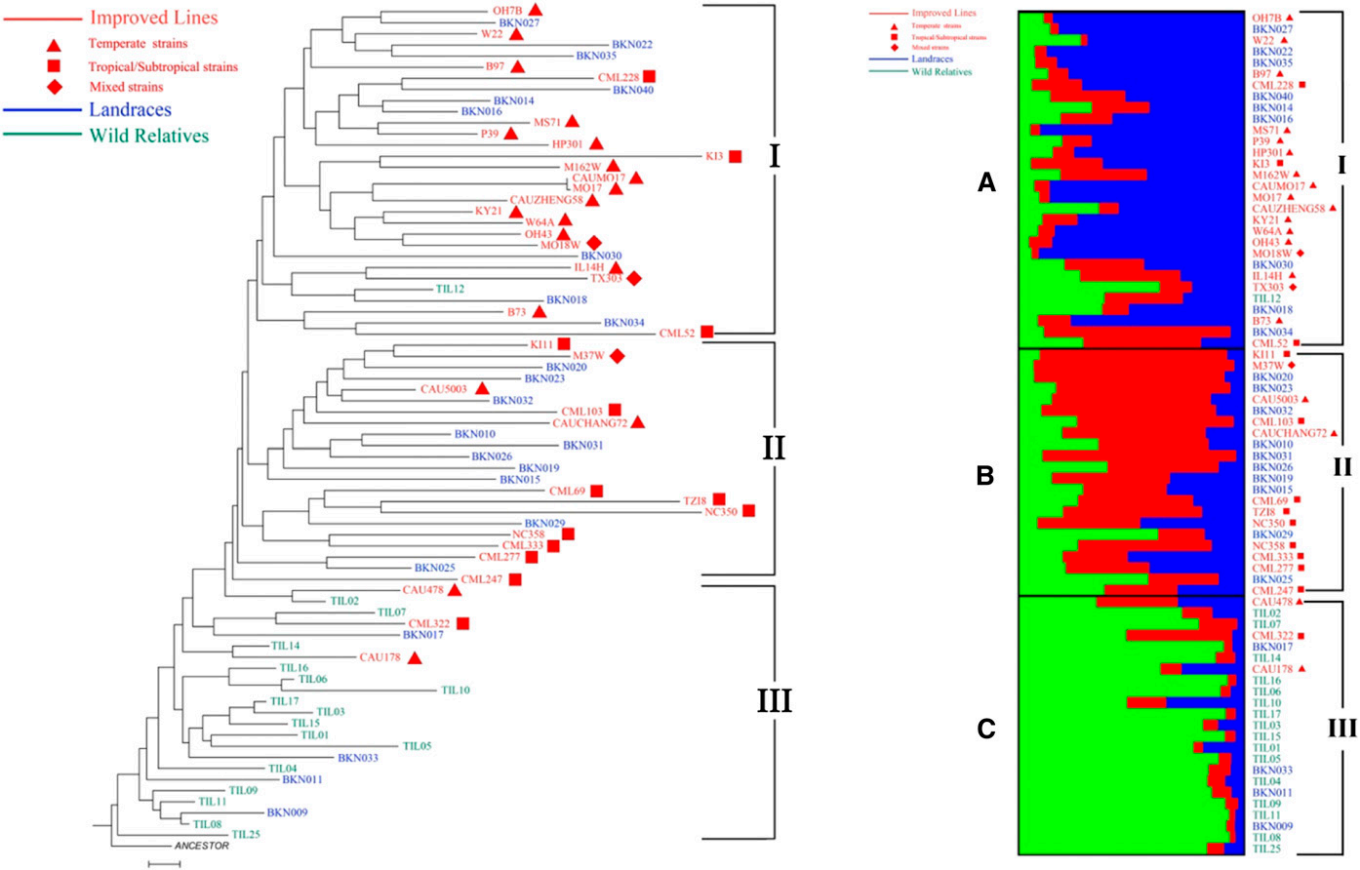
Phylogenetic relationships inferred from insertion polymorphism of *Wukong* elements in 75 maize inbreds and their relatives. (A) The phylogenetic tree of 75 inbred lines, comprising 35 improved maize lines, 23 maize landraces, and 17 wild relatives, was constructed with NJ method by PAUP*. (B) Population structure of 75 inbred lines with k = 3 that was constructed by STRUCTURE. The three populations are indicated by red, green, and blue. Three groups deduced by the N-J tree are also shown on the right side of the lines.

After excluding 29 elements that are present in all investigated strains, a total of 51 *Wukong* elements, 16 from B73 and 35 from the other 25 parents of the NAM population, were selected for the phylogenetic analysis in 75 maize inbreds and their relatives (Table S5). A neighbor-joining phylogenetic tree was then constructed based on the presence or absence matrix formed by allelic variations. The 75 accessions can be grouped into three clusters (I–III; Figure 6). Specifically, the majority of temperate and tropical inbred lines were grouped with part of the landraces into cluster I and cluster II, respectively. However, all teosintes but one were grouped into cluster III, with a small number of accessions from the other groups. The cases in which accessions from modern lines and landrace group were intermingled in clusters I and II could be explained by the fact that modern lines were selected from landraces only approximately 100 years ago (Yamasaki *et al.* 2005). Therefore, it is not unexpected to accumulate enough variation to distinguish crops themselves from the others. Still, we could successfully distinguished teosintes from the other groups, and the tropical group from the temperate group, even among different accessions of the same group (Figure 6). These results further underlined that the *Wukong* family could be reliably used as molecular markers. Moreover, we performed a population structure analysis using the same data set to validate the proposed phylogeny. When the number of populations (k) was set at three, the three groups corresponded with the data from the phylogenetic analysis, lending further support to our phylogenetic analysis.

### Identification of a putative autonomous element

**Figure 7:**
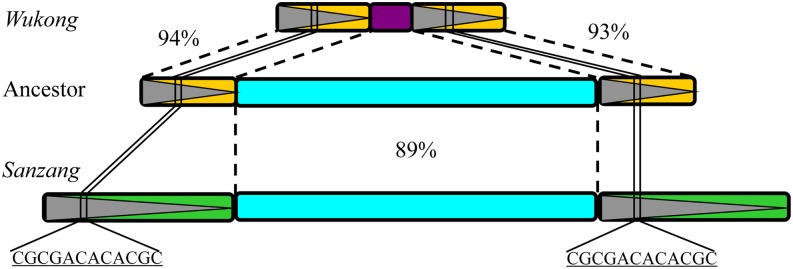
Structures of *Wukong* and its autonomous counterpart *Sanzang* element. Boxes with a triangle and the boxes in the center represent LTRs and internal region of the LTR retrotransposons, respectively. The region of similarity (94% and 93%) in LTRs between *Wukong* and the intermediate retrotransposon and the similarity (89%) in the internal region between the intermediate retrotransposon and *Sanzang* are indicated by dashed lines. The conserved 12-bp motif of LTR is indicated by solid lines.

**Figure 8:**
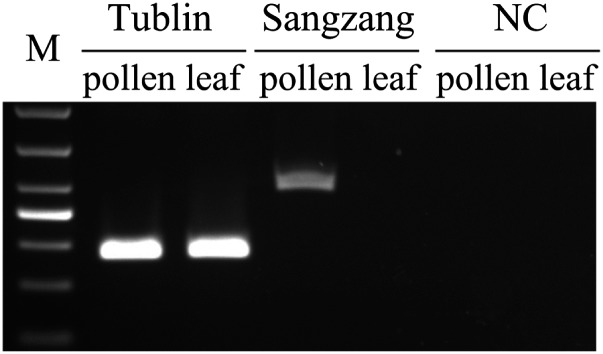
RT-PCR analysis of the autonomous retrotransposon, which was identified as the counterpart of TRIM. RT was performed on three batches of independently isolated RNAs from B73 pollen and leaf. A maize *Tublin* gene and PCR without cDNA (NC) were used as a positive control and a negative control, respectively. M represents molecular marker.

In the absence of any coding sequences, *Wukong* elements are nonautonomous retrotransposons and retrotransposition needs to be activated *in trans* by other autonomous elements. To identify such an element with pairing specificity, *Wukong* sequences were subjected to a Blast analysis of the sequenced B73 maize genome. We could identify a copia-like LTR retrotransposon on chromosome 5 (position 176,635,103-176,637,023), which could have given rise to *Wukong* elements through internal deletions because they share more than 90% identity in their LTRs and the internal domain of *Wukong* elements (Figure 7). Because this retrotransposon does not encode a functional polyprotein, we queried its sequence against the maize EST database to search for a transcript of a related copia-like element. We could identify such an mRNA sequence (NM_001156956.1), which shares approximately 90% identity with the queried sequence in the internal region, albeit with no significant similarity to both LTRs of the *Wukong* elements (Figure 7). However, based on the EST sequence data, a putative autonomous retrotransposon on chromosome 8 (position 138,704,041-138,705,912) could be identified in the maize genome and was named *Sanzang*. The *Sanzang* element has all the features of an autonomous element with a length of 1882 bp, containing two 502-bp LTRs and an 878-bp intact internal region (Figure 7). The transcription of *Sanzang* was validated by RT-PCR and found in maize pollen, but not in leaf tissue (Figure 8), suggesting that retrotransposition activity possibly could be regulated through epigenetic modification. As already considered for MITEs and SINEs that also lack sequences present in their potential autonomous controlling elements (Dewannieux *et al.* 2003; Yang *et al.* 2009), we also suggest the same for *Sanzang* because one can envision how *Wukong* elements haven arisen from the autonomous element in a series of recombination events.

## Discussion

### *Wukong*: the high recombination efficiency TRIM

TRIM elements were originally discovered in plants (Witte *et al.* 2001), have been found in almost all vascular plants (Kalendar *et al.* 2008), and have been found in ants (*Pogonomyrmex barbatus*) (Zhou and Cahan 2012). Like other LTR retrotransposons, the majority of TRIMs typically possess two LTRs (Witte *et al.* 2001). In addition, Rosaceae genomes, including pear, apple, peach, and mei, have a significant number of TRIMs that contain multiple LTRs (Yin *et al.* 2014). Moreover, TRIMs with multiple LTRs vary greatly among different species, even if the size of the genomes does not. For instance, there are 252 *Cassandra* elements with multiple LTRs present in the pear genome, whereas there are only 13 in the apple genome, although both species have approximately the same genome size. Interestingly, a higher density of elements in the genome was thought to have a benefit (10 copies/Mb in pear and 3 copies/Mb in apple) (Yin *et al.* 2014). Like the *Cassandra* elements in Rosaceae species, there are also *Wukong* elements in maize with multiple LTRs (Figure 1B, Table S2). Although the majority of them possess TSDs, others do not (Table S2). To some extent, our results support the notion that elements with multiple LTRs probably resulted from recombination between two adjacent intact elements (Yin *et al.* 2014). Still, the total copy number and density of *Wukong* elements in maize (215, 0.1 copies/Mb) were far lower than those of the *PbCassandra* elements in pear (5052, 10 copies/Mb). On the contrary, the ratio of the elements with multiple LTRs to the total number of *Wukong* elements (8%) was slightly higher than for *PbCassandra* (5%) (Yin *et al.* 2014). Therefore, the density of elements in the genome is probably not the main factor affecting formation of elements with multiple LTRs. Instead, the recombination frequency of the elements was likely more affected by their own characteristics and genomic background. Therefore, we propose that the *Wukong* element family probably possessed higher recombination efficiency compared to the *Cassandra* elements in the Rosaceae genomes. Moreover, *Wukong* elements are the first TRIM element family that possesses many members with multiple LTRs in larger genome sizes.

### *Wukong* is a promising marker for maize diversity and evolution

Considering the high abundance and dispersion, combined with the known ancestral states, TRIMs have been demonstrated to be an ideal molecular marker for investigating evolutionary relationship and genetic diversity among species. In the tribe of the Brassiceae, four TRIMs members have been used as clade markers to infer the taxonomic lineage based on insertion site polymorphisms (Yang *et al.* 2007). TRIMs in apple also have been perfectly applied to IRAP or REMAP analyses for inferring their genetic relationships among apple cultivars (Antonius-Klemola *et al.* 2006). Thanks to the NGS dataset of the Hapmap2 project (Chia *et al.* 2012), we were able to investigate the insertion polymorphism of 80 *Wukong* members, which were identified from the parents of maize NAM population (McMullen *et al.* 2009) among 75 resequenced maize inbreds and their relatives from three germplasm groups. Whereas 30 members showed monomorphic insertions in all 76 accessions, the remaining 50 insertions showed abundant insertion polymorphism among these lines (Table S5), and many of them were unique to a particular germplasm group, subgroup, or even individual lines. Moreover, these inbred lines not only could be distinguished not only from each other but also could be clustered together with members from the same group or subgroup in their phylogenetic relationships based on insertion polymorphism of TRIMs (Figure 6). These results show that *Wukong* members possess relatively high resolution as molecular markers. Regarding the CAUMo17 and Mo17 inbreds, which comprise two independent sequencing data sets for Mo17 lines, the predicted results are consistent for all detected sites, confirming their use as molecular markers (Table S5).

The phylogenetic tree generated by polymorphic *Wukong* insertions was very similar to that inferred from a large number of SNPs (Hufford *et al.* 2012). However, the expense of sequencing a large number of individual genomes remains a limitation for most population genetics studies. In comparison, the polymorphic *Wukong* insertion is a simple, cost-efficient approach for classifying the maize germplasm that only requires PCR amplification with locus-specific primers. It is doubtless that *Wukong* is a promising marker for maize diversity and evolution studies.

### *Sanzang*: the autonomous counterpart of TRIM in maize

It appears that TRIM elements have expanded in size over a relative long period of time in plant genomes (Kalendar *et al.* 2008). Moreover, several studies show that they still might be active today based on high sequence conservation, nearly identical LTRs, and the presence of TSD (Witte *et al.* 2001; Zhou and Cahan 2012). This is also the case for the *Wukong* element family. Because TRIMs have no coding capacities, they have to achieve their retrotransposition through a transacting autonomous element. Despite the evidence for recent transposition events, identification of the controlling element of TRIMs in plant genomes has been rather elusive (Witte *et al.* 2001; Zhou and Cahan 2012). In maize, however, we propose the *Sanzang* element as a potential controlling element for the *Wukong* element family. In support of this proposal, we offer the presence of a 12-bp motif residing in LTRs of both elements (Figure 7). Specifically, this motif is very conserved and could be the site for recombination of the two elements and the binding site for mobility-related proteins. Furthermore, the transcription activity of *Sanzang* in maize pollen would be consistent with this role (Figure 8). Therefore, it should be worthwhile to design experiments to test this hypothesis.

## 

## Supplementary Material

Supporting Information
