# Supplementary material for: The Wukong Terminal-Repeat Retrotransposon in Miniature (TRIM) Elements in Diverse Maize Germplasm
Source: G3 (Bethesda). 2015 May 26;5(8):1585–92. doi: 10.1534/g3.115.018317 (PMC4528315; doi:10.1534/g3.115.018317)
Supplement: Supporting Information [file supp_5_8_1585__index.html]

The Wukong Terminal-Repeat Retrotransposon in Miniature (TRIM) Elements in Diverse Maize Germplasm — Supporting Information 

# The *Wukong* Terminal-Repeat Retrotransposon in Miniature (TRIM) Elements in Diverse Maize Germplasm

## Supporting Information for Liu *et al.*, 2015

**Files in this Data Supplement:**

- Table S1 - Primers used in this study. (.xls, 44 KB)
- Table S2 - *Wukong* members identified in B73 RefGen\_v2. (.xls, 48 KB)
- Table S3 - Distributions of *Wukong* members in all chromosomes. (.xls, 24 KB)
- Table S4 - New *Wukong* members identified from the parents of NAM population. (.xls, 34 KB)
- Table S5 - Presence and absence of *Wukong* members in 76 maize and its relative lines. (.xls, 3 MB)
